# Temporal Changes in Screen-Detected Retinopathy of Prematurity Among Screened Very-Low-Birth-Weight Infants Born Before 32 Weeks of Gestation: A Single-Center Retrospective Cohort Study

**DOI:** 10.3390/children13091173

**Published:** 2026-08-31

**Authors:** Yifan Li, Hui Wu, Xuefeng Feng, Haiping Li, Hua Zhang, Yanxia You, Tongyan Han

**Affiliations:** 1 Department of Pediatrics, Peking University Third Hospital, Beijing 100191, China; 2 Department of Ophthalmology, Peking University Third Hospital, Beijing 100191, China; 3 Research Center of Clinical Epidemiology, Peking University Third Hospital, Beijing, 100191, China

**Keywords:** birth weight, disease burden, oxygen therapy, preterm infant, retinopathy of prematurity, weight gain

## Abstract

**Highlights:**

**What are the main findings?**

- Screen-detected any-stage ROP increased substantially among very-low-birth-weight infants born before 32 weeks of gestation from 2021–2022 to 2023–2024.
- The period–ROP association persisted across sequential explanatory models incorporating infant maturity, early respiratory morbidity, prescreening oxygen exposure, and early weight recovery, with more frequent documented pre-plus findings but no statistically significant increase in treatment-requiring ROP or other predefined severe indicators.

**What are the implications of the main findings?**

- The increased detection of any-stage ROP may represent a higher screening burden associated with changes in the population structure of preterm infants rather than a clear increase in severe vision-threatening ROP.
- Continuous surveillance of ROP incidence, severity, oxygen management, early growth, and follow-up completeness may help guide NICU quality monitoring.

**Abstract:**

**Background/Objectives:** Retinopathy of prematurity (ROP) remains an important cause of preventable childhood visual impairment, yet recent temporal changes among very-low-birth-weight infants in China are not well characterized. This study aimed to evaluate temporal changes in screen-detected ROP among very-low-birth-weight infants born before 32 weeks of gestation. **Methods:** We conducted a single-center retrospective cohort study of very-low-birth-weight infants who underwent ROP screening at Peking University Third Hospital from 2021 to 2024. Infants were grouped by birth period, and sequential multivariable regression models and sensitivity analyses were used to assess temporal differences in ROP outcomes. **Results:** Among 614 infants assessed for eligibility, 478 screened infants with available ROP outcomes were included. Any-stage ROP increased from 34.3% in 2021–2022 to 64.6% in 2023–2024 (*p* < 0.001). The association remained statistically significant in the sequential explanatory Model 6 (OR 2.82, 95% CI 1.73–4.60; *p* < 0.001). Treatment-requiring ROP showed a nonsignificant numerical increase from 5.1% to 9.3% (*p* = 0.112). Among infants with ROP, documented pre-plus findings were more frequent in 2023–2024 than in 2021–2022 (15.5% vs. 4.4%), and the overall plus-status distribution differed between periods (*p* = 0.040); however, maximum ROP stage and treatment-requiring ROP did not differ significantly. **Conclusions:** Screen-detected any-stage ROP increased substantially in 2023–2024, with more frequent documented pre-plus findings but no statistically significant increase in treatment-requiring ROP or other predefined severe indicators.

## 1. Introduction

Retinopathy of prematurity (ROP) is a retinal vascular disorder of preterm infants and remains an important preventable cause of childhood visual impairment worldwide [1,2,3]. Although screening and timely treatment have reduced the incidence of blindness due to severe ROP in many settings, the burden of ROP varies substantially across regions and care systems. Infants born at lower gestational age (GA) and lower birth weight (BW) are most vulnerable, but ROP is also influenced by postnatal oxygen exposure, respiratory morbidity, transfusion, infection, and early growth failure [2,4,5,6].

Temporal changes in the ROP burden are clinically meaningful because they may indicate shifts in survival among high-risk infants, changes in neonatal care, or changes in screening and documentation. Population-based and network studies have reported heterogeneous trends: ROP incidence has declined or remained stable in some high-income settings, whereas single-center and national data from China suggest that ROP, particularly any-stage ROP, may increase as survival of very immature infants improves [7,8,9,10,11]. These inconsistent findings support the value of center-specific temporal analyses, especially when clinical practice and screening processes are examined in parallel.

As ROP evolves over the first postnatal weeks, the timing of exposure assessment is important in observational analyses. Some retrospective studies have evaluated postnatal exposure using variables summarized over the entire hospitalization period, such as total oxygen duration, ventilation duration, bronchopulmonary dysplasia, or length of stay. Although clinically informative, these variables may not always preserve the temporal sequence between exposure and ROP detection. Therefore, defining the time-related exposure before the first ROP screening examination may provide a more cautious approach for evaluating temporal changes. Early postnatal growth has also been incorporated into ROP risk algorithms and may reflect illness severity, nutritional status, and insulin-like growth factor-1-related vascular development [12,13,14,15,16,17].

In our unit, the ROP screening schedule, ophthalmology team, screening equipment, and unit-level oxygen saturation targets did not undergo major changes from 2021 to 2024. However, neonatal service activity and the clinical case mix changed substantially beginning in 2023. In particular, the increase in screened infants born at <28 weeks was driven predominantly by inborn rather than transferred infants, in the context of expanded antenatal registration and high-risk obstetric care. Therefore, we used this four-year cohort to examine whether the ROP burden changed over time and whether any observed change could be explained by infant maturity, early respiratory illness, prescreening oxygen exposure, and early weight recovery.

## 2. Materials and Methods

### 2.1. Study Design and Population

This single-center, retrospective cohort study was conducted in a tertiary neonatal intensive care unit from 2021 to 2024. Infants admitted during the study period with GA < 32 weeks and BW < 1500 g were assessed for eligibility. The analytic cohort included infants who underwent at least one ROP screening examination and had an available ROP outcome. Infants were excluded if they died before the first ROP screening examination, were transferred to another hospital, were discharged against medical advice, or had unavailable ROP outcomes due to incomplete screening or follow-up. Therefore, the primary analysis represents screen-detected ROP among screened infants rather than the cumulative ROP incidence among all eligible live-born or admitted infants.

Infants were classified into the early period (2021–2022) or late period (2023–2024). This grouping was chosen to summarize the observed temporal changes in ROP burden. The calendar year was also evaluated as a continuous variable in the sensitivity analysis.

### 2.2. ROP Screening, Staging, and Outcomes

Experienced ophthalmologists performed ROP screening according to the unit protocol. During the study period, the unit protocol was based on the Chinese guideline for screening retinopathy of prematurity (2014), which recommended initiating screening at 4–6 weeks of postnatal age or 31–32 weeks’ postmenstrual age [18]. ROP screening was available on one fixed day each week throughout both study periods. There were no major changes in screening criteria, weekly screening schedule, ophthalmology team, screening equipment, or unit-level oxygen saturation targets. ROP was classified according to the International Classification of Retinopathy of Prematurity, Third Edition (ICROP3) [4]. The primary outcome was any-stage ROP. The secondary outcome was treatment-requiring ROP, defined as ROP for which ophthalmologic treatment was performed.

ROP severity was further characterized using the original ophthalmologic records. For each infant, the maximum ROP stage and most posterior zone were defined according to the more severely affected eye during follow-up [4]. Aggressive ROP was recorded when documented in the ophthalmological records, including cases historically described as aggressive posterior ROP. Because detailed eye-level type 1 ROP classification according to the Early Treatment for Retinopathy of Prematurity (ETROP) criteria [6] could not be fully reconstructed for all infants from the retrospective records, we additionally defined an exploratory severe ROP composite outcome as stage 3 or higher ROP, aggressive ROP, plus disease, or treatment-requiring ROP. Because this composite combined clinically heterogeneous and potentially overlapping severity indicators, it was used only for descriptive purposes and should not be interpreted as a validated clinical endpoint or as equivalent to ETROP type 1 ROP.

### 2.3. Clinical Variables and Prescreening Exposures

Clinical data included GA, BW, sex, small for gestational age (SGA), multiple births, cesarean delivery, antenatal corticosteroid exposure, premature rupture of membranes, assisted reproduction, Apgar scores, neonatal respiratory distress syndrome (NRDS), surfactant use, postnatal intubation, invasive ventilation duration, duration of oxygen therapy, hemodynamically significant patent ductus arteriosus, severe intraventricular hemorrhage, sepsis, pneumonia, necrotizing enterocolitis (NEC), erythropoietin exposure, transfusion, and postnatal corticosteroid use. Antenatal corticosteroid exposure was defined as any documented prenatal corticosteroid exposure in neonatal records. Postnatal intubation was defined based on the recorded clinical variables for intubation after birth or early stabilization. Transfusion variables in the descriptive analyses were based on red blood cell transfusion records, whereas temporally restricted transfusion variables were used only when the first transfusion occurred before the first ROP screening examination. NEC was defined according to the documented clinical diagnosis in medical records, and detailed Bell staging was not uniformly available in this retrospective dataset.

To reduce reverse causality, the time-dependent exposures used in explanatory models were restricted to the period before the first ROP screening examination whenever timing information was available. To account for differences in the observation time before the first screening, the proportion of prescreening oxygen exposure was calculated as the prescreening oxygen duration divided by the time from birth to the first ROP screening examination.

### 2.4. Early Weight Recovery

Early weight recovery was assessed using day-14 weight change from birth, calculated as [(day-14 weight − birth weight)/birth weight] × 100%. We also evaluated day-14 weight gain velocity and failure to regain birth weight by day 14 in the sensitivity analyses.

### 2.5. Statistical Analysis

Continuous variables were presented as median (interquartile range [IQR]) or mean (standard deviation), as appropriate. Categorical variables were presented as numbers/total numbers (percentages). Between-period comparisons were performed using the Mann–Whitney U test, chi-square test, or Fisher’s exact test, as appropriate.

Sequential logistic regression models were used to estimate the association between the late study period and any-stage ROP. Model 1 included only the study period. Model 2 was additionally adjusted for perinatal background factors, including maternal age, assisted reproduction, antenatal corticosteroid exposure, multiple births, cesarean delivery, sex, and SGA. Model 3 was additionally adjusted for GA and BW. Model 4 was additionally adjusted for NRDS and postnatal intubation. Model 5 was additionally adjusted for prescreening oxygen duration. Model 6 was additionally adjusted for day-14 weight change from birth.

These sequential models were intended as exploratory explanatory models rather than as a formal causal mediation analysis. Variables introduced in the later models, particularly respiratory morbidity, prescreening oxygen exposure, and early postnatal weight change, may partly lie on pathways linking differences in population structure and neonatal clinical course across study periods to ROP. Therefore, changes in the study-period coefficient across models were interpreted descriptively as changes in the association after accounting for these clinical characteristics, rather than as estimates of an independent or controlled direct causal effect of calendar period. All 478 infants had complete data for all covariates included in Models 1–6; therefore, no infants were excluded from these models because of missing covariate data.

We performed several sensitivity analyses. First, age at the first ROP screening examination was added to the final model. Because postmenstrual age at first screening is mathematically related to GA and postnatal age at screening, GA and postmenstrual age at first screening were not included simultaneously in the same model. Second, the proportion of prescreening oxygen exposure was used as an alternative to the absolute prescreening oxygen duration. Third, because any-stage ROP was common, a modified Poisson regression model with robust variance was fitted to estimate the adjusted relative risk. Fourth, given the limited number of treatment-requiring ROP events, treatment-requiring ROP was analyzed descriptively and using simplified Firth logistic regression models. We additionally evaluated concordance with an alternative gestational-age-dependent first-screening window based on AAP screening guidance [5]: 31 + 0 to 31 + 6 weeks’ postmenstrual age for infants born at ≤26 + 6 weeks and 28–34 days of postnatal age for infants born at ≥27 + 0 weeks. This post hoc window analysis was distinct from the Chinese guideline used by our unit during the study period [18]. Finally, to account for within-family correlation, sibling clusters were reconstructed from the source registration records using the multiple-birth indicator together with maternal-derived neonatal naming or concordant birth-episode registration information. Infants from the same multiple pregnancy were assigned a common anonymous sibling-cluster identifier, whereas singleton infants and infants whose co-sibling was not included were treated as individual clusters. Logistic regression with sibling-clustered robust standard errors and a generalized estimating equation model with an exchangeable working correlation structure were fitted. No infant was excluded from these analyses because of missing cluster information.

## 3. Results

### 3.1. Study Population and Clinical Characteristics

**Figure 1:**
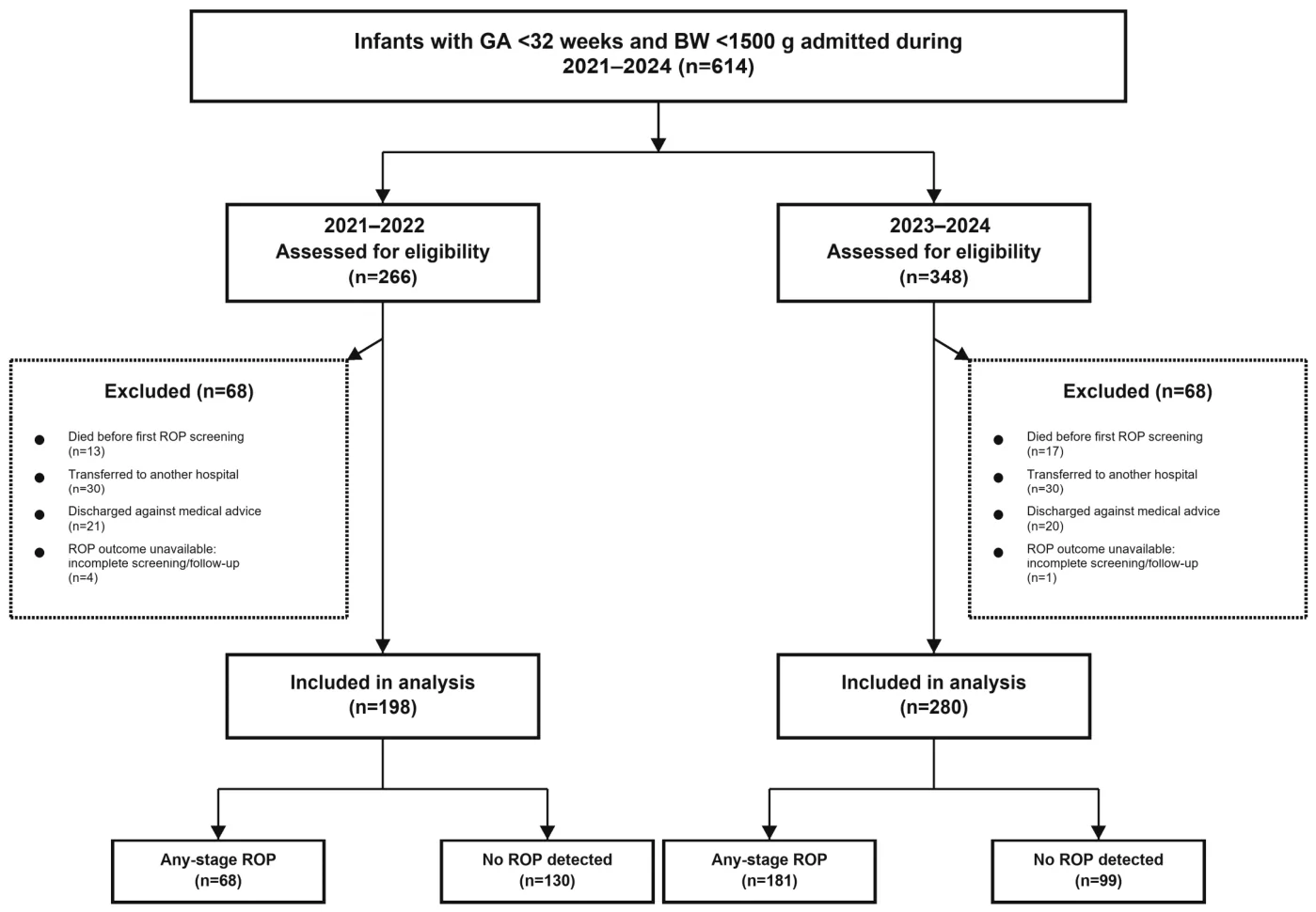
Study flow diagram. A total of 614 infants with gestational age < 32 weeks and birth weight < 1500 g were assessed for eligibility. Infants were excluded if they died before the first ROP screening examination, were transferred to another hospital, were discharged against medical advice, or had unavailable ROP outcomes because of incomplete screening or follow-up. Abbreviations: ROP retinopathy of prematurity.

A total of 614 infants with GA < 32 weeks and BW < 1500 g were assessed for eligibility during 2021–2024. Among them, 266 were admitted during 2021–2022 and 348 during 2023–2024. In 2021–2022, 68 infants were excluded, including 13 who died before the first ROP screening examination, 30 who were transferred to another hospital, 21 who were discharged against medical advice, and four whose ROP outcomes were unavailable because of incomplete screening or follow-up. In 2023–2024, 68 infants were excluded, including 17 who died before the first ROP screening examination, 30 who were transferred, 20 who were discharged against medical advice, and one whose ROP outcome was unavailable because of incomplete screening or follow-up. Finally, 478 screened infants were included in the analysis, including 198 in 2021–2022 and 280 in 2023–2024 (Figure 1).

Infants in 2023–2024 had a lower GA and BW and a higher proportion of GA < 28 weeks than infants in 2021–2022 (Table 1). NRDS and surfactant use became more common in the later period. After verification of the first-screening dates against the source records, the one-day difference in median postnatal age at first screening was not statistically significant, whereas postmenstrual age at first screening was lower in 2023–2024. Concordance with the alternative gestational-age-dependent first-screening window was similar between periods.

### 3.2. Temporal Changes in ROP Outcomes and Severity

**Figure 2:**
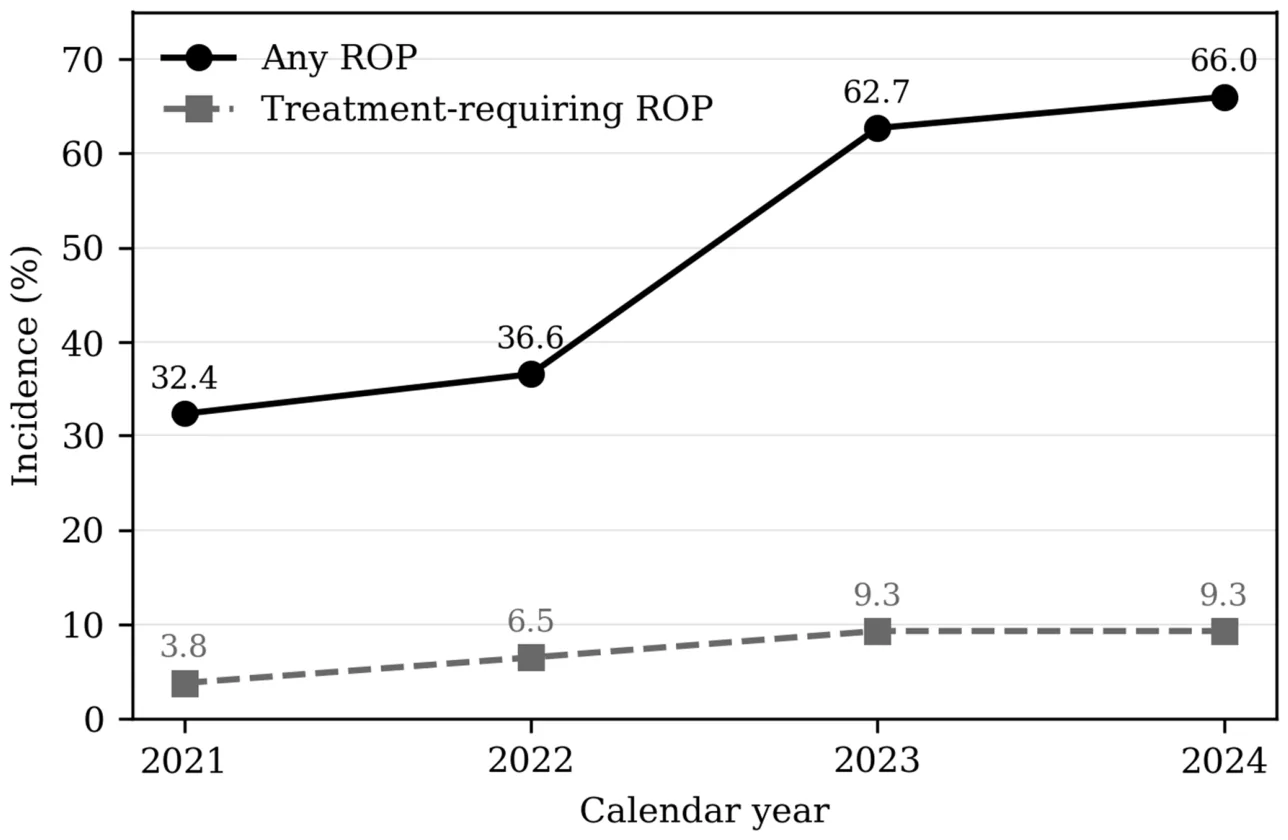
Annual incidence of any-stage ROP and treatment-requiring ROP from 2021 to 2024. Abbreviations: ROP retinopathy of prematurity.

Any-stage ROP increased from 68/198 (34.3%) in 2021–2022 to 181/280 (64.6%) in 2023–2024 (*p* < 0.001). Treatment-requiring ROP showed a nonsignificant numerical increase from 10/198 (5.1%) to 26/280 (9.3%) (Fisher’s exact test, *p* = 0.112) (Table 2). Annual incidence of any-stage ROP was 32.4% in 2021, 36.6% in 2022, 62.7% in 2023, and 66.0% in 2024. The annual proportions of screen-detected any-stage ROP were relatively similar in 2021 and 2022, followed by a marked increase in 2023 that persisted in 2024, indicating a predominantly stepwise rather than uniformly gradual change (Figure 2).

**Figure 3:**
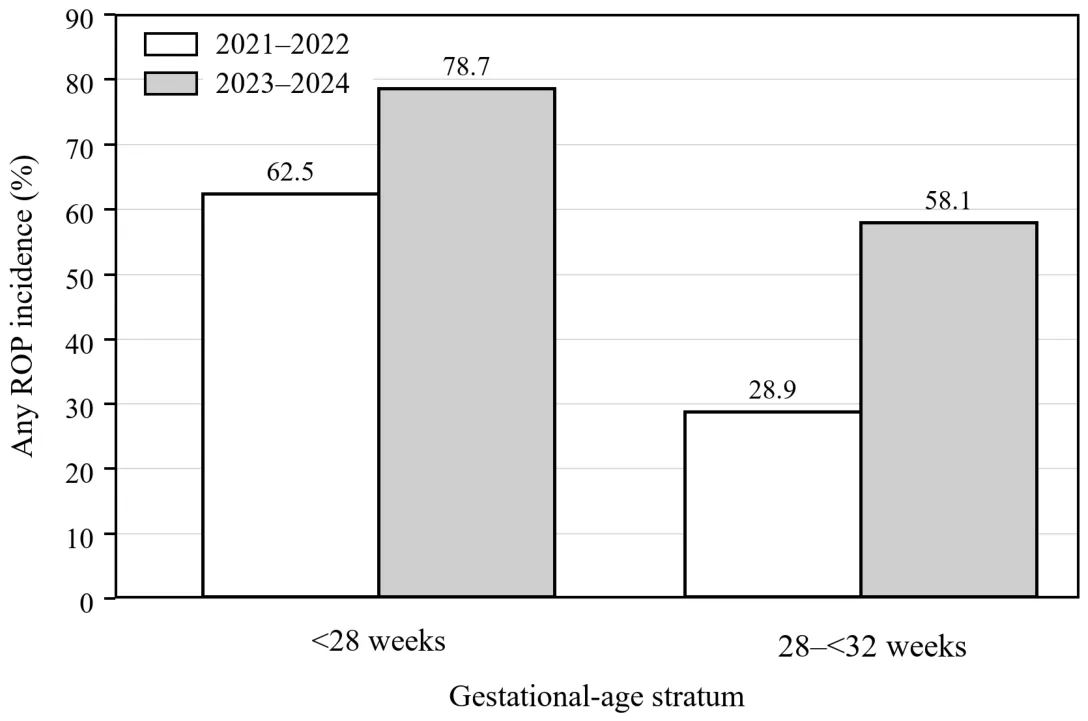
Incidence of any-stage ROP by gestational-age stratum and study period. Abbreviations: ROP retinopathy of prematurity; GA gestational age.

In the GA-stratified analyses, the incidence of any-stage ROP among infants aged <28 weeks increased from 62.5% to 78.7% (*p* = 0.073). Among infants with GA 28 to <32 weeks, any-stage ROP increased from 28.9% to 58.1% (*p* < 0.001) (Table 2 and Figure 3). The period-by-GA-stratum interaction was not statistically significant (interaction OR 0.65, 95% CI 0.24–1.73; *p* = 0.387). However, the relatively small number of infants in the <28-week stratum limited statistical power to detect potential effect modification; therefore, the nonsignificant interaction should not be interpreted as evidence that the temporal association was necessarily identical across the two GA strata.

Detailed ROP severity data were extracted from the original ophthalmological records. Among infants with ROP, the distribution of the maximum ROP stage did not differ significantly between the periods (Table 3). Stage 3 disease was observed in eight of 68 infants with ROP in 2021–2022 and in 32 of 181 in 2023–2024, whereas aggressive ROP was identified in four and three infants, respectively. Given the very small number of aggressive ROP cases (4 and 3 cases, respectively), this comparison had limited statistical power and should be interpreted cautiously. Most ROP cases in both periods involved Zone II disease. The plus-status distribution differed between periods (*p* = 0.040), driven by an increase in documented pre-plus findings from 4.4% to 15.5%, whereas plus disease did not increase. No statistically significant between-period differences were detected in the proportions of stage 3 or higher or aggressive ROP, treatment-requiring ROP, or exploratory severe ROP composite outcomes among infants with ROP. Among all screened infants, stage 3 or higher or aggressive ROP (12/198 [6.1%] vs. 35/280 [12.5%]) and exploratory severe ROP composite outcomes (14/198 [7.1%] vs. 42/280 [15.0%]) were more frequent in 2023–2024, but the associations were attenuated after adjustment for GA and BW.

### 3.3. Sequential Regression and Sensitivity Analyses

In unadjusted analysis, the late period was associated with higher odds of any-stage ROP (odds ratio [OR] 3.50, 95% confidence interval [CI] 2.39–5.12; *p* < 0.001). The association was attenuated overall from Model 1 to Model 6 but remained statistically significant in Model 6 (OR 2.82, 95% CI 1.73–4.60; *p* < 0.001) (Table 4). Covariate-specific estimates from Model 6 are shown in Supplementary Figure S1. This residual association should not be interpreted as a fully adjusted causal or controlled direct effect of study period.

After verification and correction of the first-screening dates, the median postnatal age at first ROP screening was 31.00 (IQR 28.25–33.00) days in 2021–2022 and 32.00 (IQR 29.00–34.00) days in 2023–2024 (*p* = 0.060). The corresponding postmenstrual ages were 34.00 (IQR 32.75–35.00) and 33.29 (IQR 32.25–34.61) weeks, respectively (*p* < 0.001). Concordance with the alternative gestational-age-dependent first-screening window was similar between periods (146/198 [73.7%] vs. 202/280 [72.1%]; *p* = 0.700). Any-stage ROP was detected in 175/348 (50.3%) infants screened within the window and 74/130 (56.9%) screened outside the window (*p* = 0.196). After adjustment for study period and continuous GA, window concordance was not associated with ROP (OR 0.97, 95% CI 0.62–1.54; *p* = 0.907), and the period-by-window interaction was not statistically significant (*p* = 0.864).

Given the limited number of treatment-requiring ROP events, treatment-requiring ROP events were analyzed descriptively using simplified sensitivity models. Treatment-requiring ROP occurred in 10 of 198 infants (5.1%) in 2021–2022 and in 26 of 280 infants (9.3%) in 2023–2024, representing a nonsignificant numerical increase (Fisher’s exact test, *p* = 0.112). In Firth logistic regression adjusted for GA and BW, the association between the late period and treatment-requiring ROP was not statistically significant (adjusted OR 1.36, 95% CI 0.63–2.92; *p* = 0.435). Sensitivity and exploratory analyses yielded results broadly consistent with the primary analysis (Supplementary Table S1).

## 4. Discussion

In this single-center cohort of screened very-low-birth-weight infants born before 32 weeks of gestation, screen-detected any-stage ROP increased markedly in 2023–2024 compared to 2021–2022. The association persisted across sequential explanatory models incorporating perinatal background, infant maturity, early respiratory morbidity, prescreening oxygen exposure, and early postnatal weight recovery. This was also consistent in sensitivity analyses addressing the timing of the first screening, proportional oxygen exposure, estimation of relative risk for a common outcome, and clustering among multiple births. The increase in any-stage ROP was accompanied by more frequent documentation of pre-plus findings, but not by a statistically significant increase in treatment-requiring ROP or the other predefined severe ROP indicators. These findings suggest that the observed temporal change primarily represents an increase in screen-detected ROP burden among screened infants rather than definitive evidence of a parallel increase in severe vision-threatening ROP.

The incidence of ROP varies widely across regions, neonatal care systems, and populations. Global and regional studies have shown that ROP burden is shaped not only by biological vulnerability but also by survival of extremely preterm infants, screening criteria, screening coverage, oxygen management, and follow-up systems [1,2,3,7,8,9,10,11,19,20,21]. Recent data from China also suggest that as the survival and care of very immature infants improve, the population reaching the window for retinal screening may change, and the detected ROP burden may increase in some units or regions [10,11,19,21]. Therefore, our estimates should be interpreted as screen-detected ROP among screened infants with available outcomes rather than as cumulative incidence among all live-born or admitted eligible infants.

An important contextual change around 2023 was the expansion of neonatal service activity and the high-risk obstetric population. Neonatal transfer and consultation registrations increased from 78 in 2022 to 161 in 2023, while the number of eligible infants increased from 266 in 2021–2022 to 348 in 2023–2024. Among screened infants, those born at <28 weeks increased from 32/198 (16.2%) to 89/280 (31.8%). However, the number of transferred infants born at <28 weeks increased only from 5 to 8, whereas the corresponding number of inborn infants increased from 27 to 81. Thus, the increase in extremely preterm infants was mainly attributable to inborn infants rather than to an increased number of extremely preterm transfers. The source categories remained inborn delivery and transfer from other hospitals, but their volume and case mix changed in the context of increased antenatal registration and high-risk obstetric care. These changes should be considered when interpreting the temporal increase in locally screen-detected ROP and its generalizability.

The biological plausibility of this temporal increase is supported by the central role of retinal immaturity in the pathogenesis of ROP. Lower GA remained independently associated with any-stage ROP in the final model, consistent with the concept that incomplete retinal vascularization is the substrate on which postnatal exposures act [4,22,23]. Infants in the late period were more immature and had more early respiratory morbidity, which likely contributed to the increased detected burden. Oxygen exposure is another key modifiable pathway in ROP pathogenesis; randomized trials and meta-analyses of oxygen saturation targeting in extremely preterm infants have shown that oxygen targets influence survival and ROP-related outcomes [24,25,26]. In our study, the association between the study period and any-stage ROP was attenuated but not eliminated after adjusting for prescreening oxygen duration, and it remained when proportional prescreening oxygen exposure replaced absolute oxygen duration. More detailed indicators, including fraction of inspired oxygen (FiO_2_), oxygen saturation variability, intermittent hypoxemia, and time within the target range, may be required to better characterize the quality of oxygen exposure.

Early postnatal growth provides another clinically meaningful window into ROP vulnerability. Weight-gain-based prediction models and systematic reviews have linked poor postnatal growth with severe or type 1 ROP, probably because early growth reflects nutritional accretion, systemic illness, and insulin-like growth factor-1 and vascular endothelial growth factor pathways involved in retinal vascular development [12,13,14,15,16,17]. In our cohort, infants in 2023–2024 had delayed early weight recovery and were more likely to fail to regain their birth weight by day 14. Adding day-14 weight change to the sequential explanatory model further attenuated the association between study period and ROP. However, because early postnatal growth may itself lie partly on pathways linking infant immaturity and early clinical course to ROP, this change in the study-period coefficient should not be interpreted as evidence of a direct causal effect remaining after removal of confounding. Rather, the sequential models were used to explore how measured differences in clinical characteristics may account for part of the observed temporal association. Clinically, this supports an integrated prevention perspective in which oxygen targeting, respiratory stability, infection prevention, transfusion practices, and nutritional growth are considered together rather than in isolation.

Prescreening red blood cell transfusion was less frequent in 2023–2024 than in 2021–2022 (77/280 [27.5%] vs. 92/198 [46.5%], *p* < 0.001), despite the higher frequency of screen-detected any-stage ROP in the late period. The period–ROP association persisted in a sensitivity model that included prescreening transfusion (OR 2.66, 95% CI 1.59–4.46; *p* < 0.001); prescreening transfusion itself was not statistically associated with ROP in this model (OR 0.84, 95% CI 0.50–1.40; *p* = 0.507). These results suggest that the between-period difference in transfusion exposure alone is unlikely to account for the observed temporal increase. They should not be interpreted as evidence that transfusion was protective, because transfusion exposure is related to gestational age, anemia, illness severity, phlebotomy losses, and transfusion practice, and residual confounding may remain.

The availability of ROP stage, zone, and plus-disease information allowed us to interpret the clinical meaning of the increase in any-stage ROP more carefully [4,5,6]. In particular, the very small number of aggressive ROP cases precluded a meaningful assessment of temporal differences in this uncommon severe phenotype. In our cohort, most ROP cases in both periods involved Zone II disease. Although stage 3 disease, aggressive ROP, and the exploratory severe ROP composite outcome were numerically more frequent among all screened infants in 2023–2024, these associations were attenuated after adjusting for GA and BW. Among infants with ROP, no statistically significant between-period differences were detected in the proportions with stage 3 or higher or aggressive ROP, treatment-requiring ROP, or exploratory severe ROP composite outcomes. Thus, the increase in any stage of ROP should not be interpreted as proof that the severity of the affected infants worsened. Simultaneously, treatment-requiring ROP showed a nonsignificant numerical increase, and the limited number of treatment events meant that this study was underpowered to exclude a clinically meaningful increase in treatment-requiring disease.

The more frequent documentation of pre-plus findings in 2023–2024 may represent greater retinal vascular activity. However, differences in retrospective documentation or recognition between periods, as well as chance, cannot be excluded. This finding was not accompanied by a statistically significant increase in treatment-requiring ROP or the other predefined severe ROP indicators and should therefore be interpreted cautiously.

Distinguishing between any-stage ROP and treatment-requiring or severe ROP is clinically important. Any-stage ROP is sensitive to screening timing, follow-up completeness, examiner documentation, and the detection of mild disease, whereas treatment-requiring ROP more directly reflects therapeutic burden and vision-threatening disease. Current screening guidance emphasizes timely examination, clear documentation, and completion of follow-up for infants at risk [5,18,27]. In our post hoc analysis, concordance with the alternative gestational-age-dependent first-screening window was similar between periods, and neither window concordance nor the period-by-window interaction was associated with ROP. The fixed once-weekly examination schedule may have contributed to modest deviations around the defined boundaries, but the findings do not support differential first-screening timing as an explanation for the temporal increase. Additional adjustment for postnatal age at first screening also did not materially change the period association. Nevertheless, mild disease remains particularly vulnerable to differences in screening workflows and documentation. Therefore, the observed increase is best regarded as a signal of increased local screen-detected burden and follow-up workload rather than conclusive evidence of deterioration in severe ROP outcomes.

These findings have practical implications for the quality monitoring of neonatal intensive care units. Monitoring treatment-requiring ROP alone may miss meaningful changes in the screening burden, whereas monitoring any-stage ROP alone may overstate clinical severity. A balanced local surveillance framework should include the number of eligible and screened infants, survival to first screening, screening completion, any-stage ROP, stage and zone distribution, plus disease, aggressive ROP, treatment-requiring ROP, treatment modality, and post-discharge follow-up. This is particularly relevant in settings where anti-vascular endothelial growth factor (VEGF) therapy is used because anti-VEGF treatment may be followed by persistent avascular retina or late recurrence and therefore requires structured follow-up [28,29,30,31,32]. In this study, treatment-requiring ROP was analyzed as the actual treatment received, whereas the exploratory severe ROP composite outcome was used only to describe the severity and was not considered equivalent to ETROP type 1 ROP.

This study has several strengths. It evaluated temporal changes within a single center, which reduced heterogeneity in screening criteria, ophthalmology teams, screening equipment, and unit-level oxygen saturation targets. It preferentially defined time-related exposures before the first ROP screening examination, which helped to reduce the possibility that the post-detection clinical course was misclassified as a risk factor. It also incorporated a complete eligibility flow diagram and added severity data based on the original ophthalmological records. Sensitivity analyses further addressed the first-screening timing, proportional oxygen exposure, use of odds ratios for a common outcome, treatment event sparsity, multicollinearity, and clustering among multiple births.

This study has several limitations. First, it was a retrospective, single-center study conducted in a setting where neonatal service volume and case mix changed over time, particularly through an increase in inborn extremely preterm infants; therefore, the findings may not be generalizable to centers with different obstetric populations, referral patterns, screening practices, or follow-up systems. Sufficiently detailed admission dates for a reliable monthly or quarterly incidence analysis were unavailable, limiting assessment of whether the increase around 2023 was abrupt or progressive within calendar years. Second, the analytic cohort was restricted to infants who underwent ROP screening and had available outcomes; therefore, the results reflect screen-detected ROP among screened infants rather than population-level cumulative incidence among all eligible admissions. ROP outcomes after transfer or incomplete follow-up were not fully available. Third, oxygen exposure was summarized as the duration or proportion before the first screening; detailed FiO_2_, peripheral oxygen saturation fluctuations, intermittent hypoxemia, and time-in-target-range data were unavailable. Fourth, ROP severity was retrospectively ascertained from ophthalmic records. Although stage, zone, plus disease status, and aggressive ROP were extracted, incomplete eye-specific documentation precluded consistent classification of type 1 ROP according to the ETROP criteria [6]. Examination intervals and the precise timing of regression could not be fully reconstructed. Finally, although robust and generalized estimating equation sensitivity analyses accounted for clustering among multiple births, residual within-family correlation and unmeasured confounding may remain.

## 5. Conclusions

Among screened very-low-birth-weight infants born before 32 weeks of gestation, screen-detected any-stage ROP increased substantially in 2023–2024 compared with 2021–2022, in the context of increased eligible admissions and a marked rise in inborn extremely preterm infants. The association persisted across sequential explanatory models incorporating perinatal background, infant maturity, early respiratory morbidity, prescreening oxygen exposure, and early postnatal growth. Because several of these characteristics may lie on potential causal pathways, the residual study-period association should not be interpreted as an independent causal effect of calendar period. Documented pre-plus findings were more frequent in 2023–2024, but treatment-requiring ROP and other predefined severe indicators did not show a statistically significant increase. These findings indicate an increased local screen-detected ROP burden and support continued monitoring of population structure, oxygen and respiratory care, early growth, ROP severity, treatment patterns, and follow-up completeness.

## Figures and Tables

**Table 1:** Clinical characteristics and prescreening exposures.

Characteristic	Overall (*n* = 478)	2021–2022 (*n* = 198)	2023–2024 (*n* = 280)	*p* Value
Gestational age, weeks	29.14 (27.86, 30.43)	29.71 (28.32, 30.57)	28.86 (27.68, 30.29)	<0.001
Birth weight, g	1150 (970, 1300)	1170 (1000, 1300)	1140 (930, 1292)	0.039
Maternal age, years	33 (30, 36)	33 (29, 36)	34 (30, 36)	0.023
Apgar score at 1 min	10 (8, 10)	9 (8, 10)	10 (9, 10)	<0.001
Apgar score at 5 min	10 (9, 10)	10 (9, 10)	10 (10, 10)	<0.001
Age at first ROP screening, days	31 (29, 33)	31 (28.25, 33)	32 (29, 34)	0.060
PMA at first ROP screening, weeks	33.57 (32.29, 34.86)	34.00 (32.75, 35.00)	33.29 (32.25, 34.61)	<0.001
Concordance with alternative first-screening window	348/478 (72.8%)	146/198 (73.7%)	202/280 (72.1%)	0.700
Prescreening oxygen therapy, h	720 (624, 792)	672 (480, 768)	732 (672, 792)	<0.001
Prescreening invasive ventilation, h	0 (0, 72)	0 (0, 78)	0 (0, 69.25)	0.213
Day-14 weight velocity, g/kg/day	2.14 (−1.43, 7.14)	6.43 (2.32, 11.43)	0.00 (−2.14, 3.04)	<0.001
Day-14 weight change from birth, %	2.92 (−2.10, 8.57)	7.94 (3.10, 13.83)	0.00 (−3.24, 4.36)	<0.001
Day-28 weight change from birth, %	28.08 (19.60, 36.66)	33.33 (26.03, 42.70)	24.58 (17.05, 31.05)	<0.001
Female sex	245/478 (51.3%)	98/198 (49.5%)	147/280 (52.5%)	0.517
Multiple birth	196/478 (41.0%)	81/198 (40.9%)	115/280 (41.1%)	0.972
Gestational age < 28 weeks	121/478 (25.3%)	32/198 (16.2%)	89/280 (31.8%)	<0.001
Small for gestational age	62/478 (13.0%)	25/198 (12.6%)	37/280 (13.2%)	0.850
Cesarean delivery	328/478 (68.6%)	138/198 (69.7%)	190/280 (67.9%)	0.669
Antenatal corticosteroids	167/478 (34.9%)	74/198 (37.4%)	93/280 (33.2%)	0.347
Premature rupture of membranes	104/478 (21.8%)	54/198 (27.3%)	50/280 (17.9%)	0.014
Gestational diabetes mellitus	125/478 (26.2%)	52/198 (26.3%)	73/280 (26.1%)	0.963
Assisted reproduction	144/478 (30.1%)	45/198 (22.7%)	99/280 (35.4%)	0.003
Postnatal intubation at birth/resuscitation	78/478 (16.3%)	42/198 (21.2%)	36/280 (12.9%)	0.015
NRDS	306/478 (64.0%)	93/198 (47.0%)	213/280 (76.1%)	<0.001
Pulmonary surfactant use	306/478 (64.0%)	93/198 (47.0%)	213/280 (76.1%)	<0.001
Prescreening invasive ventilation ≥ 7 days	45/478 (9.4%)	23/198 (11.6%)	22/280 (7.9%)	0.166
Prescreening transfusion	169/478 (35.4%)	92/198 (46.5%)	77/280 (27.5%)	<0.001
Day-14 failure to regain birth weight	158/478 (33.1%)	30/198 (15.2%)	128/280 (45.7%)	<0.001
hsPDA	163/478 (34.1%)	71/198 (35.9%)	92/280 (32.9%)	0.495
Sepsis	115/478 (24.1%)	56/198 (28.3%)	59/280 (21.1%)	0.069
NEC	9/478 (1.9%)	4/198 (2.0%)	5/280 (1.8%)	1.000
BPD (2018 definition)	144/478 (30.1%)	61/198 (30.8%)	83/280 (29.6%)	0.784

Data are presented as the median (interquartile range) or *n* (%). BPD, bronchopulmonary dysplasia; BW, birth weight; GA, gestational age; hsPDA, hemodynamically significant patent ductus arteriosus; NEC, necrotizing enterocolitis; NRDS, neonatal respiratory distress syndrome; PMA, postmenstrual age; ROP, retinopathy of prematurity; SGA, small for gestational age.

**Table 2:** ROP outcomes by study period and gestational-age stratum.

Outcome	Overall	2021–2022	2023–2024	*p* Value
Any ROP	249/478 (52.1%)	68/198 (34.3%)	181/280 (64.6%)	<0.001
Treatment-requiring ROP	36/478 (7.5%)	10/198 (5.1%)	26/280 (9.3%)	0.112
Any ROP among infants < 28 weeks	90/121 (74.4%)	20/32 (62.5%)	70/89 (78.7%)	0.073
Treatment-requiring ROP among infants < 28 weeks	19/121 (15.7%)	4/32 (12.5%)	15/89 (16.9%)	0.562
Any ROP among infants 28–<32 weeks	159/357 (44.5%)	48/166 (28.9%)	111/191 (58.1%)	<0.001
Treatment-requiring ROP among infants 28–<32 weeks	17/357 (4.8%)	6/166 (3.6%)	11/191 (5.8%)	0.343

**Table 3:** Distribution of ROP severity among infants with ROP by study period.

Severity Characteristic	2021–2022 ROP *n* = 68	2023–2024 ROP *n* = 181	*p* Value
Maximum ROP stage			0.141
Stage 1	15 (22.1%)	51 (28.2%)	
Stage 2	41 (60.3%)	95 (52.5%)	
Stage 3	8 (11.8%)	32 (17.7%)	
Aggressive ROP	4 (5.9%)	3 (1.7%)	
Most posterior zone			0.134
Zone I	2 (2.9%)	1 (0.6%)	
Zone II	62 (91.2%)	175 (96.7%)	
Zone III	1 (1.5%)	0 (0.0%)	
Unknown zone	3 (4.4%)	5 (2.8%)	
Plus status			0.040
No plus	55 (80.9%)	134 (74.0%)	
Plus disease	10 (14.7%)	19 (10.5%)	
Pre-plus	3 (4.4%)	28 (15.5%)	
Stage 3 or higher or aggressive ROP	12 (17.6%)	35 (19.3%)	0.857
Treatment-requiring ROP	10 (14.7%)	26 (14.4%)	1.000
Exploratory severe ROP composite outcome	14 (20.6%)	42 (23.2%)	0.735
Mild ROP	54 (79.4%)	139 (76.8%)	0.735

Data are presented as *n* (%). The exploratory severe ROP composite outcome was defined as stage 3 or higher ROP, aggressive ROP, plus disease, or treatment-requiring ROP. This exploratory variable was used for descriptive severity assessment and was not considered equivalent to ETROP type 1 ROP.

**Table 4:** Expanded sequential logistic regression models for any-stage ROP.

Model	Adjustment	*N*	OR (95% CI) for 2023–2024 vs. 2021–2022	*p* Value
Model 1	Study period only	478	3.50 (2.39–5.12)	<0.001
Model 2	Model 1 + maternal age, assisted reproduction, antenatal corticosteroids, multiple birth, cesarean delivery, sex, SGA	478	3.32 (2.24–4.91)	<0.001
Model 3	Model 2 + GA, BW	478	2.97 (1.97–4.50)	<0.001
Model 4	Model 3 + NRDS, postnatal intubation	478	3.25 (2.09–5.06)	<0.001
Model 5	Model 4 + prescreening oxygen therapy	478	3.10 (1.98–4.85)	<0.001
Model 6	Model 5 + day 14 weight change from birth	478	2.82 (1.73–4.60)	<0.001

BW, birth weight; CI, confidence interval; GA, gestational age; NRDS, neonatal respiratory distress syndrome; OR, odds ratio; ROP, retinopathy of prematurity; SGA, small-for-gestational age.

## Data Availability

The datasets generated and analyzed in the current study are available from the corresponding author upon reasonable request and subject to institutional approval.

## Supplementary Materials

The following supporting information can be downloaded at https://www.mdpi.com/article/10.3390/children13091173/s1, Figure S1: Forest plot of Model 6 of the sequential explanatory logistic regression analysis for any-stage ROP; Table S1: Sensitivity and exploratory analyses for the association between study period and screen-detected any-stage ROP.

## Abbreviations

BPD, bronchopulmonary dysplasia; BW, birth weight; CI, confidence interval; ETROP, Early Treatment for Retinopathy of Prematurity; FiO_2_, fraction of inspired oxygen; GA, gestational age; GEE, generalized estimating equation; hsPDA, hemodynamically significant patent ductus arteriosus; NEC, necrotizing enterocolitis; NRDS, neonatal respiratory distress syndrome; OR, odds ratio; PMA, postmenstrual age; ROP, retinopathy of prematurity; RR, relative risk; SGA, small for gestational age; VEGF, vascular endothelial growth factor.
