# Supplementary material for: Temporal Changes in Screen-Detected Retinopathy of Prematurity Among Screened Very-Low-Birth-Weight Infants Born Before 32 Weeks of Gestation: A Single-Center Retrospective Cohort Study
Source: Children (Basel). 2026 Aug 31;13(9):1173. doi: 10.3390/children13091173 (PMC13605036; doi:10.3390/children13091173)
Supplement: Supplementary file 1 [file children-13-01173-s001.zip › children-4491057-supplementary.pdf]

Supplementary Materials

Table S1. Sensitivity and exploratory analyses for the association between study period and screen-detected any-stage ROP.

Sensitivity analysis	Effect shown	N	Effect estimate (95% CI)	P value
Year as continuous variable	per additional calendar year	478	1.51 (1.22–1.86)	<0.001
Exploratory model using eligible univariable-significant variables	2023–2024 vs 2021–2022	478	2.86 (1.76–4.66)	<0.001
Day-14 failure to regain birth weight	2023–2024 vs 2021–2022	478	2.98 (1.87–4.74)	<0.001
Day-14 weight velocity	2023–2024 vs 2021–2022	478	2.79 (1.70–4.56)	<0.001
Plus prescreening transfusion	2023–2024 vs 2021–2022	478	2.66 (1.59–4.46)	<0.001
Plus prescreening invasive ventilation duration	2023–2024 vs 2021–2022	478	2.94 (1.79–4.84)	<0.001
Plus prescreening transfusion and invasive ventilation duration	2023–2024 vs 2021–2022	478	2.74 (1.63–4.62)	<0.001
Final model additionally adjusted for age at first ROP screening	2023–2024 vs 2021–2022	478	2.80 (1.72–4.58)	<0.001
Proportion of prescreening oxygen exposure replacing absolute oxygen duration	2023–2024 vs 2021–2022	478	2.77 (1.70–4.51)	<0.001
Modified Poisson model	2023–2024 vs 2021–2022	478	RR 1.60 (1.27–2.02)	<0.001
Firth model for treatment-requiring ROP adjusted for GA and BW	2023–2024 vs 2021–2022	478	1.36 (0.63–2.92)	0.435
Sibling-clustered robust logistic model	2023–2024 vs 2021–2022	478	2.82 (1.69–4.70)	<0.001
GEE model clustered by sibling cluster	2023–2024 vs 2021–2022	478	2.82 (1.69–4.70)	<0.001

CI, confidence interval; OR, odds ratio; RR, relative risk; ROP, retinopathy of prematurity. Unless otherwise specified, the estimates are odds ratios from logistic regression models. The modified Poisson model shows an adjusted relative risk. The exploratory model included variables that were significant in the univariate analyses and temporally available before the first screening: maternal age, assisted reproduction, cesarean delivery, gestational age, birth weight, neonatal respiratory distress syndrome, prescreening oxygen duration, prescreening invasive ventilation duration, and day-14 weight change. Robust standard-error and GEE analyses accounted for clustering among siblings from the same multiple pregnancy. Sibling-cluster information

was available for all included infants, and N=478 was retained in both analyses.

Figure S1. Forest plot of Model 6 of the sequential explanatory logistic regression analysis for any-stage ROP.

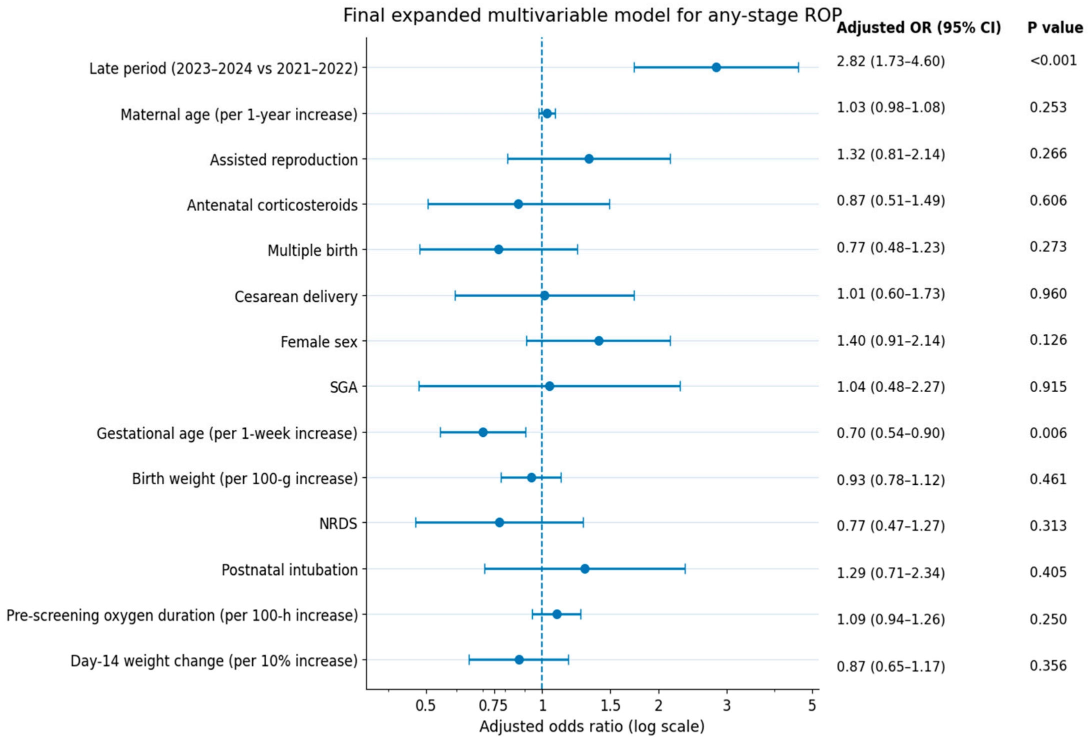

Supplementary Figure S1. Forest plot of Model 6 of the sequential explanatory logistic regression analysis for any-stage ROP. Adjusted ORs and 95% CIs are shown. Continuous variables are scaled as follows: maternal age per 1-year increase, gestational age per 1-week increase, birth weight per 100-g increase, prescreening oxygen duration per 100-h increase, and day-14 weight change per 10% increase. Abbreviations: CIs, confidence intervals; ROP, retinopathy of prematurity; OR, odds ratios.
